# Single vs. Dual Agonist Pharmacotherapy for Managing Insufficient Weight Loss and Weight Regain Following Metabolic and Bariatric Surgery: A Comparative Review

**DOI:** 10.3390/nu18040553

**Published:** 2026-02-07

**Authors:** Claudia Reytor-González, Martín Campuzano-Donoso, Gerardo Sarno, Martha Montalvan, Raquel Horowitz, Gianluca Rossetti, Vincenzo Pilone, Luigi Barrea, Giovanna Muscogiuri, Luigi Schiavo, Daniel Simancas-Racines

**Affiliations:** 1 Facultad de Ciencias de la Salud y Bienestar Humano, Universidad Tecnológica Indoamérica, Ambato 180150, Ecuador; claudiareytor@gmail.com (C.R.-G.);; 2 “San Giovanni di Dio e Ruggi D’Aragona” University Hospital, Scuola Medica Salernitana, 84131 Salerno, Italy; 3 Escuela de Medicina, Universidad Espíritu Santo, Samborondón 0901952, Ecuador; 4 Geriatrics Division, Department of Medicine, Montefiore Medical Center, 3411 Wayne Avenue, New York, NY 10467, USA; 5 General and Bariatric Surgery Unit, Abano Terme Policlinic, 35031 Padova, Italy; 6 Public Health Department, Federico II University, 80131 Naples, Italy; 7 Department of Psychology and Health Sciences, Pegaso Telematic University, 80143 Naples, Italy; 8 Endocrinology Unit, Department of Clinical Medicine and Surgery, Federico II University, 80131 Naples, Italy; 9 Italian Centre for the Care and Wellbeing of Patients with Obesity, Federico II University Hospital, 80131 Naples, Italy; 10 Cattedra Unesco Educazione Alla Salute e Allo Sviluppo Sostenibile, Federico II University, 80131 Naples, Italy; 11 Department of Medicine, Surgery and Dentistry “Scuola Medica Salernitana”, University of Salerno, 84081 Baronissi, Italy; lschiavo@unisa.it

**Keywords:** bariatric surgery, weight gain, weight loss, glucagon-like peptide 1, glucose-dependent insulinotropic polypeptide, pharmacotherapy

## Abstract

Weight management after metabolic and bariatric surgery remains a persistent clinical challenge, particularly when patients experience insufficient weight loss or progressive weight regain following the postoperative nadir. In recent years, pharmacological therapies targeting gut-derived hormones have reshaped the therapeutic approach, offering nonsurgical strategies that directly influence appetite regulation, satiety, and energy balance. Single agonists acting on the glucagon-like peptide one receptor have demonstrated meaningful reductions in body weight among postoperative patients, while dual agonists that target both the glucagon-like peptide one receptor and the glucose-dependent insulinotropic polypeptide receptor have shown even greater weight reduction in early studies, suggesting enhanced therapeutic potential. These benefits, however, must be interpreted within the unique anatomical, nutritional, and behavioral context of individuals who have undergone metabolic and bariatric procedures, as they are inherently at higher risk for micronutrient deficiencies, gastrointestinal intolerance, and maladaptive eating patterns. Successful treatment requires a balanced integration of pharmacotherapy, individualized nutritional guidance, psychological support, and a patient-centered model of long-term care. Although emerging evidence is promising, dedicated clinical trials are still needed to directly compare the efficacy, safety, and sustainability of single versus dual agonist therapies in postoperative populations. Furthermore, culturally sensitive dietary strategies and shared decision-making processes are essential to enhance adherence, optimize long-term outcomes, and ensure equitable access to treatment. Ultimately, these therapies represent a significant advance in addressing postoperative weight challenges, but their full potential will rely on comprehensive, multidisciplinary frameworks that support both biological and behavioral aspects of chronic weight management.

## 1. Introduction

Recognized as a global health crisis, obesity is strongly associated with cardiovascular disease, type 2 diabetes, and numerous other comorbidities [1,2]. Current clinical guidelines recommend metabolic and bariatric surgery (MBS) as a central therapeutic option for patients with a body mass index (BMI) ≥35 kg/m^2^, or for those with a BMI of 30–34.9 kg/m^2^ who present with metabolic complications [3].

The terminology surrounding these interventions has also evolved over time. The concept of “metabolic surgery” was first introduced in 1972 by H.W. Scott to describe the metabolic benefits of ileal bypass in improving hypercholesterolemia and arteriosclerosis [4]. More recently, Rubino and colleagues proposed the term “metabolic bariatric surgery” in 2016, reflecting both the weight-dependent and weight-independent mechanisms of modern procedures. This shift highlights a growing recognition that these surgeries extend beyond purely restrictive or malabsorptive classifications, encompassing metabolic effects [5].

MBS remains the most effective and durable intervention for individuals with severe obesity and related comorbidities [5]. While outcomes vary across procedure types, meta-analyses demonstrate that Roux-en-Y gastric bypass (RYGB) produces an average total body weight loss (TBWL) of 25–30% at one year, compared with approximately 20–25% after sleeve gastrectomy (SG) [6]. For patients with higher BMI, more complex procedures such as biliopancreatic diversion with duodenal switch (BPD/DS) and its streamlined variant, single-anastomosis duodenal-ileal bypass with sleeve gastrectomy (SADI-S), have shown sustained long-term effectiveness. A recent cohort study with at least 60 months of follow-up reported that both procedures achieved >20% total weight loss in the vast majority of patients (96% for BPD/DS and 91% for SADI-S), with comparable remission rates of obesity-related comorbidities [7]. Beyond weight reduction, these interventions are highly effective in inducing remission or significant improvement of obesity-related conditions, including type 2 diabetes, hypertension, and dyslipidemia, leading to substantial improvements in cardiometabolic health and quality of life [5,8].

Despite these transformative outcomes, weight recidivism remains a major challenge following MBS [9]. This phenomenon, manifesting either as insufficient weight loss (IWL) or subsequent weight regain (WR), threatens to reverse the health benefits of surgery, leading to recurrence of comorbidities and a decline in psychological well-being [10,11,12]. Estimates suggest that 20–30% of patients either fail to achieve expected weight loss targets or experience significant WR over time, with prevalence varying by procedure type and length of follow-up [13]. Such figures underscore the need for ongoing strategies to sustain surgical benefits.

Historically, therapeutic avenues for addressing IWL and WR were limited. Revision surgery was often considered but carries heightened risks, while earlier generations of anti-obesity medications (AOMs) offered modest and inconsistent benefits [14]. The landscape has shifted dramatically with the introduction of incretin-based pharmacotherapies, which target the gut–brain axis to regulate appetite, satiety, and glucose metabolism [15]. These agents, particularly glucagon-like peptide-1 receptor agonists (GLP-1 RAs) and the newer class of dual agonists (GLP-1/glucose-dependent insulinotropic polypeptide (GIP) agonists), offer a non-surgical, mechanism-driven approach to mitigating weight recidivism [16]. Their incorporation into multidisciplinary care represents a paradigm shift, expanding the therapeutic toolkit available to clinicians managing post-bariatric patients [15].

Recognizing the chronic nature of obesity and the limitations of surgery alone, major societies now endorse pharmacotherapy as a key pillar of long-term care. The American Society for Metabolic and Bariatric Surgery (ASMBS) and the International Federation for the Surgery of Obesity and Metabolic Disorders (IFSO) recommend anti-obesity medications, particularly GLP-1 receptor agonists, as a critical adjunctive treatment for patients experiencing IWL or WR after MBS [3,17]. These guidelines reinforce that pharmacological intervention is not a temporary fix but a necessary component of a lifelong, multidisciplinary strategy to sustain surgical benefits and manage obesity as a chronic, relapsing disease [3].

This review will explore the evolving role of pharmacotherapy in this context, with a particular focus on comparing single-agonist (GLP-1 RA) and dual-agonist (GLP-1/GIP) therapies. We will examine their mechanisms of action, summarize the current clinical evidence on efficacy and safety in post-bariatric populations, and highlight nutritional, behavioral, and patient-centered considerations that influence treatment success. Ultimately, this analysis aims to clarify whether single or dual agonists hold the upper hand in addressing IWL and WR, while situating pharmacotherapy as part of a holistic, long-term care strategy that extends beyond surgery. Given the limited availability of randomized or comparative trials evaluating incretin-based therapies in post-bariatric populations, this review does not aim to establish therapeutic superiority. Rather, it synthesizes available post-bariatric evidence and integrates mechanistic and non-surgical obesity data to inform clinical decision-making and identify knowledge gaps.

## 2. Methodology

This narrative review was conducted through a structured literature search of PubMed, Scopus, and other relevant biomedical databases from inception through 2025. Search terms were combined using Boolean operators and included “bariatric surgery,” “metabolic surgery,” “weight regain,” “insufficient weight loss,” “GLP-1 receptor agonist,” “dual incretin agonist,” “GIP,” “semaglutide,” and “tirzepatide.” Priority was given to studies evaluating post-bariatric populations; however, when direct evidence was limited, high-quality trials in non-surgical obesity populations were included to contextualize findings. In addition, reference lists of included articles and relevant reviews were manually screened, and grey literature (conference proceedings and society statements) was reviewed when appropriate. Data extraction was performed independently by multiple reviewers, and any discrepancies were resolved by consensus discussion. Given the heterogeneity and limited availability of randomized trials in post-bariatric cohorts, a narrative synthesis approach was adopted rather than a formal systematic review.

## 3. Understanding Weight Recidivism Post-Bariatric Surgery

For clarity, IWL and WR are defined here using commonly accepted clinical thresholds, while acknowledging variability across the literature. Weight recidivism following MBS has emerged as one of the most challenging barriers to achieving durable outcomes [13]. It encompasses two interrelated but distinct phenomena: IWL and WR. IWL is generally defined as failure to achieve ≥50% excess weight loss (EWL) or <20% TBWL within the first 1–2 years after surgery, while WR refers to the progressive increase in body weight following the initial attainment of nadir weight [18,19]. Although definitions vary across studies, both conditions are clinically relevant, as they compromise the long-term metabolic and quality-of-life benefits of surgery [10].

The prevalence of IWL and WR is influenced by the type of procedure, length of follow-up, and patient-specific characteristics [18]. After sleeve gastrectomy (SG), WR rates have been reported as 5.7% at 2 years, rising to nearly 40% by 5 years [20,21], with some systematic reviews citing figures as high as 76% over extended follow-up [21]. RYGB appears somewhat more protective, though not immune, with WR estimates ranging from 3.9% at 3–7 years to 15–27% at 5 years [18,22]. Laparoscopic adjustable gastric banding (LAGB), which has largely fallen out of favor, demonstrates the poorest long-term durability, with 35–40% of lost weight often regained and a prevalence approaching 38% at 10 years [23]. These figures highlight the chronic, relapsing nature of obesity even in the context of surgical intervention.

The etiology of weight recidivism is multifactorial, reflecting a complex interplay of physiological, behavioral, psychological, and nutritional determinants [24]. From a physiological standpoint, surgery induces profound but not permanent alterations in appetite-regulating hormones [25]. Early reductions in ghrelin and increases in GLP-1 and peptide YY (PYY) enhance satiety and limit intake [26]. However, longitudinal studies suggest that these hormonal benefits may diminish, with patients who experience WR demonstrating persistently elevated ghrelin and blunted postprandial GLP-1 responses compared with weight maintainers [27,28]. Such hormonal adaptations reflect the body’s intrinsic tendency to defend its highest sustained weight, contributing to increased hunger and caloric intake over time.

Anatomical factors may further affect surgical efficacy. Enlargement of the gastric pouch, dilation of the gastric sleeve or outlet, or the formation of a gastro-gastric fistula after RYGB can expand gastric capacity, reducing restriction and facilitating increased caloric intake [29,30]. Though less common than behavioral factors, these anatomic changes represent potentially correctable contributors to recidivism, often requiring revisional procedures if clinically significant [31].

Behavioral drivers are perhaps the most pervasive contributors. Maladaptive eating behaviors, including grazing, loss-of-control eating, and binge eating, have been repeatedly linked to WR [22,32,33]. Grazing alone has been reported in 17–47% of patients after surgery and shows a strong correlation with long-term recidivism [33]. Although the mechanical limitations imposed by MBS may prevent the large-volume binges characteristic of preoperative binge eating disorder, loss of control over food intake remains clinically relevant and predictive of poor outcomes [34]. These behaviors are often compounded by a return to calorie-dense diets, sugary beverages, and a sedentary lifestyle, which collectively undermine the metabolic advantages conferred by surgery [35].

Psychological and emotional health are closely intertwined with these behaviors. Depression, anxiety, stress, and poor coping mechanisms have all been associated with impaired adherence to dietary and physical activity recommendations [36]. Emotional eating, in particular, creates a feedback loop in which distress drives consumption, further promoting WR and worsening psychological well-being [37]. These findings highlight the need for ongoing mental health evaluation and support as an integral component of long-term bariatric care [24].

Nutritional considerations add another critical aspect. Many patients enter surgery with pre-existing deficiencies, most notably in vitamin D and iron, which can be exacerbated by restrictive or malabsorptive procedures [38]. Long-term, patients remain at risk for deficiencies in iron, vitamin B12, folate, calcium, and fat-soluble vitamins (A, D, E, and K) [38]. Inadequate protein intake and chronic micronutrient insufficiency not only impair metabolic health but may also contribute indirectly to WR by reducing satiety, inducing fatigue, and promoting sarcopenia [39]. Thus, nutritional decline can act both as a medical complication and as a behavioral risk factor for recidivism [39,40].

Adherence to lifelong supplementation and dietary guidelines is therefore essential, yet it remains a persistent challenge. Barriers include forgetfulness, cost, pill burden, gastrointestinal (GI) intolerance, and limited engagement from healthcare providers [40]. Surveys consistently reveal patient dissatisfaction with the education and follow-up they receive regarding supplementation, suggesting that non-adherence is often less about patient neglect and more about gaps in multidisciplinary care delivery [41]. This underscores the importance of individualized education, structured follow-up, and culturally sensitive strategies to improve compliance.

**Figure 1:**
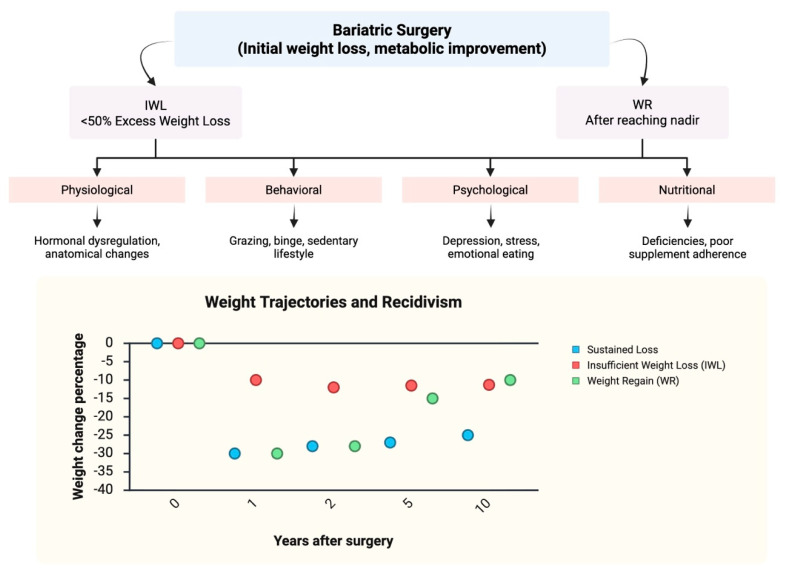
Illustrative Conceptual Model of Weight-regain Patterns After MBS. The figure summarizes common long-term weight trajectories after MBS and the multifactorial contributors to insufficient weight loss (IWL) and weight regain (WR). The top panel depicts two suboptimal outcome patterns: IWL, defined as failure to achieve ≥50% excess weight loss within 1–2 years, and WR, characterized by progressive weight increase after reaching nadir weight. These patterns reflect the interaction of physiological, behavioral, psychological, and nutritional factors. The graph presents illustrative long-term trajectories of weight change after surgery, showing that while most patients achieve substantial and durable reductions, a subset experience IWL, represented by a flattened weight-loss curve, or WR, characterized by progressive regain after nadir. Reported prevalence varies by procedure and duration of follow-up, with WR affecting up to 40% of patients at 5 years after sleeve gastrectomy and even higher rates documented following laparoscopic adjustable gastric banding in long-term cohorts [7,17,18,19,24,39]. This figure is intended to provide conceptual context for post-MBS weight recidivism and does not depict the pharmacologic mechanisms of incretin-based therapies, which are addressed separately in the text and tables.

Taken together, IWL and WR are not simply failures of surgical technique but rather the result of a dynamic interaction between biological adaptation, anatomy, behavior, mental health, and nutrition [18]. Recognizing weight recidivism as a predictable and multifactorial process reframes the problem from surgical “failure” to one of chronic disease management [42]. Addressing it requires not only careful patient selection and surgical expertise but also long-term, multidisciplinary follow-up that integrates medical, nutritional, psychological, and behavioral strategies to sustain the benefits of surgery over a lifetime. Figure 1 provides a conceptual overview of weight recidivism after bariatric surgery, illustrating the definitions and multifactorial causes of IWL and WR, alongside representative long-term weight trajectories observed in clinical practice.

## 4. Mechanisms of Action: A Comparison of Incretin Agonists

Incretin-based pharmacotherapy has revolutionized obesity management, offering a mechanism-driven approach that complements the physiological changes in MBS [43,44]. These drugs harness the actions of gut-derived hormones that regulate satiety, glucose metabolism, and energy balance [45]. Two major classes are currently at the forefront: single agonists targeting the GLP-1 RAs and dual agonists that engage both GLP-1 and glucose-dependent insulinotropic GIP receptors [45]. Understanding their distinct mechanisms provides insight into their therapeutic potential in addressing IWL and WR following MBS (Table 1).

### 4.1. Single Agonists (GLP-1 Receptor Agonists)

The first generation of effective incretin-based therapies is GLP-1 RAs, represented by agents such as liraglutide and semaglutide, which are administered as subcutaneous injections using standardized dose-escalation schedules to improve tolerability. Liraglutide is typically administered once daily, with gradual titration to a target dose of 3.0 mg, whereas semaglutide is administered once weekly, with stepwise escalation to a target dose of 2.4 mg in obesity management [46]. These compounds are synthetic analogs of the endogenous hormone GLP-1, secreted by intestinal L-cells in response to nutrient ingestion [47]. By activating GLP-1 receptors expressed in multiple organs, including the hypothalamus, hindbrain, pancreas, and GI tract, these agents exert pleiotropic effects relevant to weight and metabolic control [46].

Their primary mechanisms of action include:

Central appetite regulation: GLP-1 RAs act on neural circuits within the hypothalamus and brainstem to suppress hunger, enhance satiety, and reduce food cravings, leading to sustained caloric restriction [51].

GI effects: By delaying gastric emptying, these agents prolong fullness and blunt postprandial glucose excursions, contributing to both weight reduction and metabolic stability [46].

Glycemic control: GLP-1 RAs enhance glucose-dependent insulin secretion from pancreatic β-cells while simultaneously suppressing inappropriate glucagon secretion from α-cells, thereby improving glycemic control without a significant risk of hypoglycemia [51].

Beyond weight loss, these drugs provide cardiovascular and renal protection, making them valuable in patients with obesity and coexisting metabolic disease [47]. However, despite their substantial efficacy, limitations remain: the degree of weight loss can plateau, and some patients, particularly those with severe post-surgical weight recidivism, may not achieve or sustain their desired outcomes with GLP-1 monotherapy [55].

### 4.2. Dual Agonists (GLP-1/GIP Receptor Agonists)

The next step in incretin pharmacotherapy is represented by dual agonists, such as tirzepatide, which simultaneously target the GLP-1 and GIP receptors [53]. Tirzepatide is usually administered as a once-weekly subcutaneous injection, with stepwise dose escalation from a low starting dose to maintenance doses typically ranging from 5 to 10–15 mg, depending on tolerability and clinical response [53]. GIP, secreted by K-cells in the proximal small intestine in response to dietary fat and carbohydrates, was traditionally considered less therapeutically relevant than GLP-1 [51,56]. However, recent studies have revealed that its combined use with GLP-1 signaling produces synergistic and more potent metabolic effects [54].

The mechanisms of action of dual agonists are the following:

Enhanced appetite and satiety signaling: Both GLP-1 and GIP influence central pathways regulating hunger and satiety, and their concurrent activation amplifies anorectic effects, leading to greater reductions in energy intake compared with GLP-1 RAs alone [50,54,57].

Complementary effects on adipose tissue metabolism: While GLP-1 promotes lipolysis through sympathetic activation, GIP supports healthy adipocyte function by improving lipid storage efficiency in subcutaneous fat, thereby reducing ectopic fat accumulation in liver and muscle [58]. This dual effect improves insulin sensitivity and overall metabolic health [51].

Balanced glucose regulation: GIP enhances insulin secretion during hyperglycemia and, in the context of hypoglycemia, stimulates glucagon release, while GLP-1 suppresses glucagon during hyperglycemia. This counter-regulatory interplay provides a more physiologically balanced approach to glycemic control [54].

Clinical studies suggest that this intricate synergy translates into greater and more durable weight loss than with GLP-1 RAs alone, alongside robust improvements in glycemic parameters [53]. Tirzepatide, for example, has consistently produced higher weight reduction compared with semaglutide in non-surgical populations, fueling interest in its application among post-bariatric patients who face the dual challenges of weight recidivism and metabolic relapse [59].

Together, these pharmacological advances mark a paradigm shift in the management of obesity. While GLP-1 RAs have set a new standard of care, dual agonists may offer theoretical or mechanistic advantages, offering enhanced efficacy through more comprehensive engagement of the incretin system.

## 5. Clinical Evidence in Post-Bariatric Populations

Since the introduction of incretin-based therapies, clinical and observational evidence has been accumulating regarding their use in patients who have undergone MBS and now struggle with IWL or WR. This section reviews what is known so far, both strengths and gaps, regarding efficacy, safety, nutritional impact, and procedural modifiers of response.

### 5.1. Efficacy in Addressing IWL and WR

The available data, though more limited than in non-surgical obesity populations, generally support the conclusion that GLP-1 RAs can produce clinically meaningful weight reductions in post-bariatric patients [60]. A systematic review and meta-analysis of five studies (three RCTs and two non-RCTs) evaluating weight loss in patients with obesity found that semaglutide and liraglutide, and the co-agonist efinopegdutide, were all well-tolerated and associated with significant weight loss compared to placebo. Among the four studies directly comparing semaglutide to liraglutide, three showed semaglutide to be significantly more effective for weight reduction. The meta-analysis of these four studies revealed an overall significantly greater effect on mean weight loss with semaglutide versus liraglutide (SMD: −6.39, 95% CI: −9.40, −3.38). Conversely, when semaglutide (−7.51% reduction) was compared to efinopegdutide (−8.58% reduction), no significant difference in weight loss was found [61]. Another systematic review and meta-analysis synthesized data from twelve RCTs to assess the impact of liraglutide on weight. It reported that patients with obesity treated with liraglutide achieved a mean weight loss of 3.35 kg and a corresponding reduction in their BMI of 1.45 kg/m^2^ [62].

These results are reflected in real-world observational studies. For example, a retrospective, single-arm study in Chile analyzing the use of liraglutide (median dose of 1.2 mg) in post-bariatric surgery patients found that it was effective and safe for weight reduction in routine clinical care. Mean weight loss from baseline at 3, 6, 12, 24, and 36 months ranged from 5.0% to 7.7%, with a mean body mass index reduction of 14.8% at 36 months [63]. Similarly, a retrospective cohort study in Saudi Arabia evaluating liraglutide 3.0 mg in patients with obesity over six months reported a mean weight loss of 6.5 kg (*p* < 0.001), with 52.6% of subjects achieving a ≥5% weight loss, and also demonstrated significantly better glycemic control [64]. In a real-world US cohort study, semaglutide at 1.7 mg or 2.4 mg doses for patients with overweight or obesity achieved TBWL percentages of 5.9% at 3 months and 10.9% at 6 months, suggesting an effectiveness comparable to that seen in randomized clinical trials [65].

A more recent observational study in Switzerland combining liraglutide and semaglutide over 12 months reported a median TBWL of 10.5 kg, corresponding to a mean BMI reduction of 3.7 kg/m^2^ and recovery of ~99% of the weight regained. In subgroup analysis, semaglutide appeared more effective than liraglutide (BMI reduction 4.7 vs. 3.1 kg/m^2^; *p* = 0.04). Adverse events were mild and transient, reported in about 32.5% of patients [66].

It is worth noting that cessation of GLP-1 therapy tends to be followed by weight re-gains, regardless of treatment duration [67]. A Phase 3 randomized withdrawal trial confirmed the critical need for sustained therapy for long-term weight management. Participants with obesity or overweight who stopped the GLP-1/GIP co-agonist tirzepatide after achieving an initial 20.9% mean weight loss regained a substantial 14.0% of that weight over 52 weeks. Conversely, those who continued tirzepatide were able to maintain their achieved loss and realize an additional 5.5% mean weight reduction during the follow-up period [68]. Another randomized withdrawal trial investigated the effect of sustained therapy in adults with overweight or obesity who had achieved a mean 10.6% weight loss during an initial 20-week run-in on semaglutide 2.4 mg. Over the next 48 weeks, participants who continued semaglutide achieved an additional mean −7.9% weight loss, whereas those switched to placebo experienced a substantial mean +6.9% WR, confirming that sustained treatment is necessary for continued weight reduction [69].

While the evidence for GLP-1 monotherapy is relatively mature, data comparing single to dual agonists in the post-bariatric context are still emerging [70]. One retrospective cohort of patients with weight recurrence after sleeve gastrectomy compared semaglutide vs. tirzepatide over 6 months. In that study, tirzepatide produced a mean weight loss of 15.5% from pre-treatment baseline, significantly greater than the 10.3% observed with semaglutide (*p* < 0.02). However, this finding must be interpreted cautiously, as it stems from a single, small retrospective study in post-sleeve patients. While the difference is significant, it suggests a potential trend rather than definitive superiority, pending confirmation in robust RCTs. This suggests that dual agonists may offer a more potent initial effect, though head-to-head trials remain few and follow-up is short [71].

In non-surgical obesity populations, tirzepatide has demonstrated robust weight-loss efficacy; a meta-analysis of RCTs reported consistent superiority over comparators, with many participants achieving ≥15% weight reduction [72]. This provides a plausible rationale for investigating tirzepatide (and other dual agonists) more aggressively in post-bariatric populations. Indeed, pilot trials are underway (e.g., the GRABS study) to evaluate tirzepatide for persistent obesity after surgery [73].

The distinction between direct post-bariatric evidence and extrapolated findings from general obesity trials highlights the limited but promising data for tirzepatide in this unique population (Table 2). Overall, the data support that incretin-based pharmacotherapy can recapture a substantial fraction of regained weight in many post-bariatric patients. The magnitude of benefit appears greater when initiated earlier, with more potent agents, and when combined with behavioral support, but we still lack large randomized trials in surgical cohorts to firmly define treatment algorithms.

### 5.2. Safety, Tolerability, and Nutritional Impact

The safety profiles of both GLP-1 and dual agonists have been well characterized in broader obesity and diabetes trials; however, post-bariatric patients present unique anatomical and nutritional vulnerabilities that must be considered [75].

#### 5.2.1. Gastrointestinal Side Effects and Tolerability

As in non-surgical populations, the most common adverse events are GI, including nausea, vomiting, diarrhea, and constipation [76]. In post-bariatric patients, altered GI anatomy (e.g., reduced gastric reservoir, faster transit) can exacerbate these effects, so gradual dose titration is especially important [77,78]. Some studies suggest that GI intolerance may be more frequent or severe in this group, requiring slower escalation or lower maximal doses [79].

#### 5.2.2. Nutrient Intake, Protein, and Micronutrient Risks

Because these agents suppress appetite and reduce caloric intake, they may exacerbate already precarious nutritional status in post-bariatric individuals [55,60]. Reduced food volume can limit protein intake, and in patients with marginal reserves, this may precipitate or accelerate muscle mass loss (sarcopenia) [80]. Additionally, patients are already at high risk of deficiencies in iron, vitamin B12, folate, vitamin D, calcium, and fat-soluble vitamins due to surgical malabsorption or exclusion [81]. The further limitation of intake may intensify these risks.

Furthermore, inadequate micronutrient absorption or supplementation non-adherence may result in anemia, osteopenia, neuropathy, or hepatic dysfunction [81]. Clinicians must maintain vigilant surveillance through frequent laboratory testing and adjust supplementation regimens proactively [81]. Some authors argue that baseline nutritional status should be optimized before initiating pharmacotherapy, and that more aggressive supplementation may be warranted in patients receiving GLP-1 or dual agonists [79].

#### 5.2.3. Other Safety Concerns

Although uncommon, GLP-1 receptor agonists have been infrequently associated with pancreatitis and gallbladder disease in clinical trials and post-marketing surveillance, and with thyroid C-cell hyperplasia in animal models; however, causal relationships in humans remain unproven and overall absolute risks appear low [82,83,84,85]. Importantly, evidence for these rare events is derived largely from non-surgical populations, and specific data in post-bariatric cohorts are sparse. Similarly, in tirzepatide trials, adverse event profiles mirror those of GLP-1 RAs, primarily GI in nature; discontinuation rates increase with dose [86]. In a dose–response analysis, Kasagga et al. showed that participants on higher tirzepatide doses were more likely to achieve ≥15% weight loss but also had higher rates of adverse symptoms [74].

Because post-bariatric patients often have altered renal, hepatic, and GI function, pharmacokinetics may differ, and long-term safety data in this subgroup remain limited [87]. Until more evidence accumulates, clinicians should monitor for GI intolerance, nutritional deterioration, and signs of micronutrient deficiency more aggressively than in non-surgical patients [81,86].

### 5.3. Influence of Surgical Procedure and the Hierarchy of Benefits

#### 5.3.1. Procedure-Dependent Response Variability

The type of bariatric surgery performed appears to influence both the magnitude of benefit from pharmacotherapy and the risks of complications [88,89]. Patients who had RYGB face more profound malabsorptive changes and anatomical rearrangements, which may heighten sensitivity to GI side effects or nutritional risks with incretin therapy [90]. Intestinal bypass may alter drug absorption or first-pass metabolism, though empirical data are scarce [91].

In contrast, individuals who underwent SG retain more physiological continuity of the GI tract. Because GLP-1 RAs work primarily through appetite and satiety pathways, SG patients may derive greater relative benefit from these agents, with generally lower malabsorption risks [79,92]. Some observational reports suggest better tolerability and somewhat greater percent weight loss in SG than in bypass patients, though head-to-head data are limited [93].

#### 5.3.2. Hierarchy of Benefits: Surgery and Pharmacotherapy

It is crucial to situate pharmacotherapy within the broader spectrum of obesity interventions [94]. A recent comparative analysis using real-world data found that MBS achieved a mean total weight loss of ~28.3% over 2 years, compared to ~10.3% for GLP-1 RA therapy in a matched cohort (*p* < 0.001) [95]. Another large cohort study comparing surgery vs. GLP-1 RAs in obese patients with diabetes reported that surgery was associated with lower mortality (adjusted hazard ratio ~0.38 in those with ≤10 years of diabetes) and reduction in major adverse cardiovascular events, mediated in part by greater weight loss [96]. These findings suggest that while GLP-1 or dual agonists are powerful adjuncts, they generally do not exceed the effects of surgery itself on weight, metabolic outcomes, and survival benefit. This reinforces the view that pharmacotherapy is best conceptualized as a complement, not a substitute, to surgical intervention, especially in patients with suboptimal surgical response.

## 6. Nutritional and Behavioral Considerations

The long-term success of adjunctive pharmacotherapy in post-bariatric patients hinges critically on embedding the drug regimen within a robust, patient-centered nutritional and behavioral framework [70]. In essence, pharmacotherapy should be viewed not as a standalone solution but as a facilitator that amplifies and sustains behavioral and nutritional strategies tailored to the altered physiology of post-surgical patients [97,98].

In addition to the well-recognized nutritional risks faced by post-bariatric patients, the profound appetite suppression induced by GLP-1 and dual agonists further heightens the risk of sarcopenia and loss of metabolically active lean mass [79]. To maintain optimal body composition, patients require structured nutritional and physical activity interventions that deliberately counteract medication-induced reductions in intake [79]. Evidence underscores the importance of prioritizing protein intake after bariatric surgery, as a longitudinal study of banded RYGB patients showed that protein intake dropped from ~80 g/day preoperatively to ~45 g/day at 1 month and remained inadequate at 6 months, when only 25% met ≥60 g/day, while lower fat-free mass loss was linked to higher physical activity rather than protein intake, likely due to uniformly low protein consumption in the cohort [99]. Adequate protein consumption, generally 60–100 g/day or approximately ≥1.5 g/kg ideal body weight, should be prioritized to support muscle protein synthesis, while targeted resistance training performed 2–3 times weekly remains the most effective strategy to preserve lean mass and physical function [100]. Because reduced oral intake occurs in the context of surgical-induced malabsorption, clinicians must also intensify micronutrient surveillance, monitoring iron, vitamin B12, folate, vitamin D, and calcium at least every 6–12 months and initiating prompt, high-dose supplementation when deficiencies arise to prevent long-term complications [40,101].

A cornerstone of this integrated model is nutritional guidance by a trained dietitian. Given the substantial reduction in gastric capacity and possible malabsorption inherent in MBS, patients must adopt sustainable dietary behaviors that align with both their altered anatomy and the appetite-suppressing effects of incretin therapies [79,102]. Key recommendations include prioritizing protein intake (often 60–100 g per day, depending on individual factors) to preserve lean body mass and support satiety, maintaining optimal hydration, and avoiding high-sugar, high-fat, and ultra-processed foods that may provoke cravings or contribute to relapse [79,81]. Professional dietitians can also guide patients in adopting mindful eating techniques, using portion control, and learning to interpret new satiety cues in a surgically altered gut [97].

Because post-bariatric patients already face increased lifelong risk of macro- and micronutrient deficiencies, the additional suppression of appetite and reduced intake resulting from incretin agonists raises the stakes for vigilant, long-term nutritional monitoring [103]. Routine laboratory surveillance should include indices of iron status, vitamin B12, folate, calcium, and fat-soluble vitamins (A, D, E, K), among others [79,88]. The ASMBS guidelines endorse regular supplementation (multivitamin, B12, iron, calcium + vitamin D) for all bariatric patients, with doses adjusted based on laboratory findings. Failure to adhere to supplementation can lead to severe complications, including anemia, osteoporosis, neurological deficits, and protein malnutrition [104,105].

Yet adherence to supplementation is often suboptimal, with patients citing barriers such as cost, pill burden, gastrointestinal side effects, and inadequate provider support [106]. These practical challenges must be proactively addressed through patient education, simpler formulations (e.g., chewable or liquid versions initially), and close follow-up to troubleshoot tolerability issues [107].

Behavioral and psychological support is equally indispensable. Pharmacotherapy can blunt metabolic drives toward hunger, but it cannot resolve the underlying psychological, emotional, and behavioral drivers that often precipitate weight recidivism [108,109]. Disordered eating behaviors (grazing, emotional eating, loss-of-control eating), stress-related eating, and lapses in physical activity must be confronted with structured behavioral interventions [24,110]. Cognitive-behavioral therapy (CBT), motivational interviewing (MI), acceptance-based approaches, and self-monitoring techniques are key tools in this arsenal [111].

In a pilot study of an acceptance-based behavioral intervention deployed in bariatric patients with WR, a 10-week program reversed weight gain (mean TBWL of ~3.6%) and improved psychological flexibility and eating behaviors [112]. Similarly, a remotely delivered acceptance-based intervention (Project HELP) produced a mean 5.1% weight loss (±5.5%) over the intervention period, maintained at 3-month follow-up [113]. A systematic review and meta-analysis of 124 randomized trials evaluating Behavioral Weight Management Programs (BWMPs) found that despite common WR, these interventions significantly reduce cardiometabolic risk factors, with beneficial effects persisting for at least five years after the program ends. However, the evidence is less certain regarding the BWMPs’ long-term impact on actually reducing the incidence of cardiovascular disease or type 2 diabetes itself, and the risk factor improvements were observed to dwindle as weight was regained [114].

In a multicenter randomized trial of telephone-based CBT delivered one year after bariatric surgery, short-term weight outcomes did not differ significantly between intervention and control groups; however, eating disorder symptoms (emotional and binge eating) and psychological distress (depression, anxiety) improved significantly [115]. The trial authors suggest that these psychological gains may mediate longer-term differences in weight trajectory as follow-up continues beyond the short term [115]. A similar study evaluating telephone-based CBT in post-bariatric surgery patients found significant short-term reductions in binge eating, emotional eating, anxiety, and depressive symptoms, with improvements in emotional eating lasting up to 12 months. However, despite these psychological benefits, the 7-session Tele-CBT protocol did not result in a significant difference in weight loss trajectories between the intervention and control groups over the 18-month follow-up period [116]. These findings emphasize that behavioral interventions may not always produce immediate weight changes but can exert protective effects against relapse by stabilizing psychological and eating patterns over time.

Another critical insight is that the weight-lowering benefits of incretin agonists are not durable once treatment is discontinued [55]. According to multiple studies, patients typically regain approximately two-thirds of the lost weight within a year of stopping therapy [117,118]. This rebound also erodes improvements in cardiometabolic parameters [45,118]. These observations reinforce the conceptualization of incretin agonists as long-term or possibly lifelong therapies for obesity, which creates practical and public health challenges given high costs, access limitations, and potential interruptions in supply [119]. A systematic review of 155 randomized trials investigating BWMPs found that at the conclusion of the program, participants had achieved an average of 2.8 kg greater weight loss compared to control groups. The analysis concluded that BWMPs demonstrating this pattern of initial loss and subsequent regain would still be considered cost-effective if delivered for less than £560 per person [120].

Given this, the role of behaviorally based relapse prevention becomes paramount. Behavioral strategies such as regular self-monitoring of weight, dietary journaling, periodic reinforcement or “boosters,” and early detection of upward weight trends allow for timely intervention or escalation (e.g., adjusting medication dose, re-engaging therapy) [111,120]. These techniques have been well-established in long-term weight management research and may be adapted to the post-bariatric population, though direct evidence is limited [5,81,121].

In summary, successful integration of pharmacotherapy in post-bariatric care demands a sophisticated, multidisciplinary foundation: nutritional support tailored to reduced intake and malabsorption risk, vigilant monitoring and supplementation, and structured behavioral therapy to reinforce adherence, prevent relapse, and address psychological drivers of WR. Without this scaffolding, even the most potent pharmacologic agents may fail to deliver sustained benefit.

## 7. Patient-Centered and Cultural Perspectives

The integration of pharmacotherapy into post-bariatric care cannot be separated from the individual and cultural contexts in which patients live. Beyond physiology and pharmacology, outcomes are shaped by demographic variation, cultural dietary practices, personal values, and the quality of the therapeutic alliance [122,123]. A patient-centered approach is therefore essential to ensure both effectiveness and sustainability [124].

### 7.1. Variations in Pharmacotherapy Response Across Diverse Populations

Clinical evidence indicates that responses to incretin-based therapies are not uniform across patient populations. Demographic factors, including age, sex, and ethnicity, can influence both the magnitude of weight loss and the tolerability of treatment [125]. For example, studies suggest that older adults may experience attenuated weight loss compared to younger individuals, possibly due to age-related changes in metabolism and body composition [126,127]. On the other hand, some trials report greater relative weight reduction in women than in men under the same dosing regimens [128,129]. A meta-analysis of fourteen RCTs covering multiple GLP-1 RAs, including dulaglutide and semaglutide, found a significant sex difference in weight reduction, with females losing more weight than males (pooled difference of 0.88 kg). This study concluded that the weight reduction sex difference became more pronounced as the overall magnitude of weight loss increased, particularly when the GLP-1 RAs were indicated specifically for obesity [130]. The presence of comorbidities also plays a critical role; patients with type 2 diabetes often demonstrate smaller reductions in body weight than their non-diabetic counterparts, likely reflecting differences in baseline insulin resistance and energy metabolism [131]. These findings highlight the importance of personalized pharmacotherapy, tailoring drug selection, dosing, and follow-up to the unique demographic and metabolic profile of each patient rather than applying uniform expectations.

### 7.2. Cultural Dietary Considerations

Food is more than sustenance; it is central to cultural identity, social rituals, and family cohesion [132]. For post-bariatric patients, strict adherence to Western-style dietary prescriptions may create tension with long-standing cultural practices, leading to isolation or nonadherence [133]. For example, communal meals rich in traditional foods may conflict with restrictive dietary guidelines, producing guilt or alienation [134,135]. A culturally responsive approach acknowledges these realities, prioritizing collaboration over prescription. Rather than imposing a rigid dietary framework, clinicians and dietitians should work with patients to adapt nutritional recommendations in ways that respect cultural traditions while still advancing weight management goals [136,137]. Practical strategies may include modifying portion sizes, substituting healthier preparation methods, or identifying culturally familiar protein sources. By supporting patients in navigating social and cultural food environments, care teams can enhance adherence, reduce stigma, and foster empowerment [39].

### 7.3. Shared Decision-Making

Shared decision-making (SDM) represents the cornerstone of patient-centered care in obesity management [138,139]. In this model, clinicians provide clear, personalized information about treatment options, including their potential risks, benefits, and long-term implications, while patients articulate their preferences, goals, and concerns [139]. For pharmacotherapy, where long-term adherence may be necessary, and side effects can affect quality of life, SDM is especially critical. This collaborative process helps align treatment strategies with the patient’s values, builds trust, and ensures that expectations are realistic [140]. Evidence shows that patients engaged in SDM are more likely to adhere to treatment regimens, report higher satisfaction, and may offer better outcomes [138]. In the context of incretin-based therapies, SDM allows clinicians to weigh issues such as cost, tolerability, and patient lifestyle alongside efficacy, ultimately supporting informed and sustainable choices [141].

### 7.4. Multidisciplinary Strategies to Optimize Outcomes

Obesity is a chronic, multifactorial disease, and its successful management requires the expertise of a coordinated, multidisciplinary team [142]. Pharmacotherapy is most effective when integrated into a continuum of care that also includes the input of bariatric surgeons, endocrinologists, dietitians, pharmacists, psychologists, and behavioral therapists [143]. This collaborative model ensures that treatment addresses not only physiological drivers of WR but also nutritional, behavioral, and psychosocial dimensions [144]. For example, a dietitian may tailor macronutrient distribution to mitigate deficiencies, while a behavioral therapist works with the patient to overcome maladaptive eating patterns. Endocrinologists can oversee pharmacologic titration, and pharmacists can provide guidance on drug–drug interactions or supplement compatibility. Multidisciplinary care has been consistently associated with improved long-term outcomes in post-bariatric populations, reducing the risk of nutritional complications, enhancing adherence, and strengthening relapse prevention [142,145].

## 8. Conclusions and Future Directions

Weight recidivism following MBS remains a pressing challenge, with IWL and WR undermining the long-term health benefits of surgery. Incretin-based pharmacotherapy, particularly GLP-1 RAs and dual GLP-1/GIP receptor agonists, has emerged as a powerful adjunctive strategy capable of addressing these gaps. Dual agonists are mechanistically promising for enhanced weight loss; however, direct evidence in post-MBS populations remains limited to low-level, predominantly retrospective studies. This necessitates cautious interpretation of comparative efficacy. With head-to-head evidence currently lacking, making treatment choices remains dependent on established single-agent response and clinical judgment. Importantly, pharmacotherapy should not be viewed as a replacement for surgery, but rather as a complementary intervention within a comprehensive, multidisciplinary care model.

Safety and tolerability in the post-MBS setting largely mirror findings from non-surgical populations, with gastrointestinal side effects being the most frequent concern. However, altered anatomy and reduced intake heighten the risk of nutritional deficiencies, making vigilant monitoring, supplementation, and dietary counseling essential. The integration of pharmacotherapy with behavioral therapy, nutritional support, and long-term psychological care is paramount to maximize efficacy and mitigate relapse. Furthermore, patient-centered and culturally sensitive approaches, including shared decision-making and adaptation of dietary guidance to cultural contexts, are indispensable to improving adherence and sustaining benefits.

An important practical implication is that the weight-reducing benefits of incretin-based therapies are not maintained after treatment discontinuation, with consistent evidence of substantial weight regain in withdrawal studies. This reinforces the conceptualization of obesity as a chronic disease and suggests that, for many patients, pharmacotherapy will need to be maintained long term to sustain benefits. This reality raises important challenges related to cost, access, long-term safety, and adherence, which must be addressed in future clinical and health-system research.

Looking forward, several critical directions require emphasis. First, large-scale randomized controlled trials specifically targeting post-bariatric populations are urgently needed to clarify the comparative effectiveness of single versus dual agonists, optimal timing of initiation, and long-term safety. Second, real-world implementation studies should evaluate cost-effectiveness, accessibility, and adherence barriers, particularly in health systems where medication costs and supply constraints limit sustained use. Third, future pharmacotherapy may expand to include triple agonists (GLP-1/GIP/glucagon) and other next-generation metabolic agents, which hold the potential to further augment weight loss and improve cardiometabolic outcomes. Finally, research should prioritize integrative strategies that embed pharmacotherapy within multidisciplinary, culturally responsive frameworks to ensure durable results and equitable care across diverse populations.

In conclusion, incretin-based therapies represent a paradigm shift in the management of IWL and WR after MBS. By combining pharmacological innovation with individualized nutritional, behavioral, and psychosocial support, clinicians can move closer to realizing the full and lasting benefits of bariatric surgery in patients living with obesity.

## Figures and Tables

**Table 1:** Mechanistic and clinical characteristics of single vs. dual incretin agonists in obesity and post-bariatric populations.

	Single Agonists (GLP-1 RAs: Liraglutide, Semaglutide, etc.) [46,47,48,49,50,51]	Dual Agonists (GLP-1/GIP RAs: Tirzepatide, etc.) [48,51,52,53,54]
Origin & Mechanism	Synthetic analogs of GLP-1; act on GLP-1 receptors in brain, pancreas, and gut.	Synthetic peptides activating both GLP-1 and GIP receptors.
Primary Sites of Action	Hypothalamus & brainstem (satiety), pancreas (insulin/glucagon), gut (gastric emptying).	Same as GLP-1 plus adipose tissue (lipid metabolism), enhanced central satiety pathways.
Effects on Appetite & Satiety	Strong appetite suppression, reduced cravings, prolonged satiety.	Greater anorectic effect via synergistic central signaling, leading to more pronounced calorie restriction.
Gastrointestinal Effects	Slows gastric emptying, reduces postprandial glycemia.	Similar gastric effects, with additional benefits from GIP in nutrient sensing.
Glycemic Control	Enhances glucose-dependent insulin secretion; suppresses glucagon during hyperglycemia.	Adds balanced glucagonotropic action: GLP-1 suppresses glucagon in hyperglycemia; GIP supports glucagon release in hypoglycemia, providing tighter glucose regulation.
Effects on Adipose Tissue	Indirectly promotes lipolysis via sympathetic activation.	GLP-1 promotes lipolysis + GIP improves lipid storage in subcutaneous fat, reduces ectopic fat deposition, improves insulin sensitivity.
Cardiometabolic Benefits	Proven CV risk reduction (liraglutide, semaglutide); renal protective effects.	Emerging evidence of cardiometabolic benefit; long-term CV outcome trials underway.
Weight Loss Efficacy (non-surgical populations)	Liraglutide: ~5–8% TBWL; Semaglutide 2.4 mg: ~12–15% TBWL.	Tirzepatide: ~15–22% TBWL in SURMOUNT trials—has shown greater weight loss efficacy to semaglutide.
Evidence in Post-Bariatric Patients	Increasing real-world and trial data showing benefit in IWL and WR; semaglutide most studied to date.	Limited but promising early data; ongoing studies expected to clarify efficacy and safety specifically post-MBS.
Safety & Tolerability	GI side effects common (nausea, vomiting, diarrhea); generally manageable. Rare risks: pancreatitis, gallstones.	Similar GI profile; some reports of slightly higher rates but well tolerated overall. Long-term safety data still accumulating.
Impact on Micronutrient Absorption	Minimal direct effect, but reduced oral intake may exacerbate deficiencies in MBS patients.	Same as GLP-1; indirect impact rather than direct malabsorption.
Dosing & Administration	Daily (liraglutide) or weekly (semaglutide, dulaglutide) injections.	Weekly subcutaneous injection (tirzepatide).
Limitations	Weight loss plateaus in some patients; not effective for all.	Clinical use currently limited to tirzepatide; long-term comparative data post-MBS lacking.
Relative Maturity of Post-Bariatric Evidence (Narrative Assessment) *	Moderate to High ^1^	Low to Moderate ^2^
Future Directions	Potential oral formulations; ongoing trials in bariatric cohorts.	Expansion of dual agonists and next-generation triple agonists (GLP-1/GIP/glucagon).

* These ^1^ Moderate-High: it means there are multiple restrospective and observational studies, and real-world data. ^2^ Low-Moderate: it means there are no dedicated randomized trials, and limited post-bariatric subgroup analyses.

**Table 2:** Summary of evidence: post-bariatric vs. non-surgical trials.

Pharmacotherapy	Direct Evidence in Post-Bariatric Patients	Extrapolated Evidence (Non-Surgical Obesity Trials)
Liraglutide	A retrospective study in Chile showed mean weight loss of 5.0% to 7.7% over 3–36 months [63]. A Swiss study found it less effective than semaglutide (BMI reduction of 3.1 vs. 4.7 kg/m^2^) [66] A Saudi cohort reported a mean weight loss of 6.5 kg over 6 months [64]	A meta-analysis of 12 RCTs showed a mean weight loss of 3.35 kg and BMI reduction of 1.45 kg/m^2^ [62] Established as an early standard but generally shows lower efficacy ceilings compared to newer agents [67]
Semaglutide	In a retrospective cohort, patients achieved ~10.3% weight loss over 6 months [71]. Demonstrated significantly greater mean weight loss compared to liraglutide in meta-analyses of post-surgical patients [61].	Clinical trials report ~12–15% total body weight loss (TBWL) with the 2.4 mg dose [65]. A withdrawal trial showed that patients who switched to placebo after 20 weeks regained mean +6.9% of weight, confirming the need for chronic use [69].
Tirzepatide	A retrospective cohort of post-sleeve gastrectomy patients showed a mean weight loss of 15.5% at 6 months, significantly superior to semaglutide [71] Data is currently limited to early retrospective findings and ongoing pilot trials like the GRABS study [73]	Consistently produces superior weight reduction (~15–22% TBWL) in the SURMOUNT trials compared to comparators [59] Participants who stopped therapy after initial loss regained 14.0% of their weight over 52 weeks [59] Adverse events mirror GLP-1s but discontinuation rates increase with higher doses [74].

## Data Availability

No new data were created or analyzed in this study. Data Sharing is not applicable to this article.
